# Linking Termite Feeding Preferences and Soil Physical Functioning in Southern-Indian Woodlands

**DOI:** 10.3390/insects10010004

**Published:** 2019-01-04

**Authors:** Sougueh Cheik, Rashmi Ramesh Shanbhag, Ajay Harit, Nicolas Bottinelli, Raman Sukumar, Pascal Jouquet

**Affiliations:** 1 Indo-French Cell for Water Science (IFCWS), Civil Engineering Department, Indian Institute of Science, Bangalore 560012, India; pascal.jouquet@ird.fr; 2 Institute of Ecology and Environmental Sciences (UMR 242 iEES-Paris), Sorbonne Universités, Centre IRD, 93143 Bondy, France; nicolas.bottinelli@ird.fr; 3 Indian Plywood Industries Research and Training Institute, Bangalore 560022, India; rashmishanbhags@gmail.com; 4 School of Environmental Sciences, Mahatma Gandhi University, Kottayam 686560, India; om.harit@gmail.com; 5 Soils and Fertilizers Research Institute (SFRI), Dong Ngac, Tu Liem, Ha Noi, Vietnam; 6 Centre for Ecological Sciences, Indian Institute of Science, Bangalore 560012, India; rsuku@iisc.ac.in

**Keywords:** *Odontotermes obesus*, sheeting, termite foraging activity, litter quality, organic resource consumption, soil water dynamic

## Abstract

Termites are undoubtedly amongst the most important soil macroinvertebrate decomposers in semi-arid environments in India. However, in this specific type of environment, the influence of termite foraging activity on soil functioning remains unexplored. Therefore, this study examines the link between the quality of litter and the functional impact of termite feeding preferences on soil properties and soil hydraulic conductivity in a deciduous forest in southern India. Different organic resources (elephant dung: “ED”, elephant grass: “EG”, acacia leaves: “AL” and layers of cardboard: “CB”) were applied on repacked soil cores. ED appeared to be the most attractive resource to *Odontotermes obesus*, leading to a larger amount of soil sheeting (i.e., the soil used by termites for covering the litter they consume), more numerous and larger holes in the ground and a lower soil bulk density. As a consequence, ED increased the soil hydraulic conductivity (4-fold) compared with the control soil. Thus, this study highlights that the more *O. obesus* prefers a substrate, the more this species impacts soil dynamics and water infiltration in the soil. This study also shows that ED can be used as an efficient substrate for accelerating the infiltration of water in southern-Indian soils, mainly through the production of galleries that are open on the soil surface, offering new perspectives on termite management in this environment.

## 1. Introduction

Soil biodiversity regulates a large number of ecological functions, such as the degradation of litter, the cycling of nutrients or the regulation of water dynamics in the soil [1,2]. Amongst soil organisms, termites are increasingly recognized as playing a role in the provisioning of key ecosystem services [3,4]. Termites are ubiquitously amongst the most abundant and active litter decomposers in tropical environments [5] and are also considered significant soil bioturbators or ecosystem engineers because of the biostructures they produce [6,7]. Indeed, a large body of literature describes the specific soil biological, chemical and physical properties of termite mound nests [8,9,10,11,12,13,14,15,16,17] and the link between these properties and the ecological needs of termites (i.e., to main an homeostatic environment within their nest for the colony and the fungus to grow [18,19,20]).

However, less is known about the biostructures termites produce while they forage on the ground. Indeed, termites also build belowground galleries for foraging and translocate large quantities of soil to the surface (i.e., so-called sheetings) to cover the litter they consume (e.g., leaves, wood and herbivore dung) [7], and protect themselves from light, desiccation and predators [21,22,23,24]. A recent review revealed that sheetings have different soil properties than the surrounding top-soil, depending on the properties of the top-soil and the strategy developed by termites (i.e., favouring clay or organic matter for ensuring the stability of sheetings) [23]. Less is known, however, about the quantity of sheetings that termites produce on the ground and whether this quantity varies with the quality of the substrate termites consume [25,26]. The production of sheeting is associated with the construction of belowground galleries that are used by termites to bring litter into their nest. These galleries have a significant and positive impact on water infiltration in soil and therefore reduce the susceptibility of the soil to erosion [27,28,29], although the opposite can be observed when soil sheetings collapse and produce a structural crust on the ground, limiting water infiltration and increasing water runoff [30].

Since most of the research on the functional impact of termite foraging activity has been carried out in Africa and to a lesser extent in South-America and Australia, the aim of this research was to determine whether termite foraging activity varies with the quality of the litter in a southern-Indian forest. More precisely, this study also questioned the relationship between termite food preferences and the abundance of soil sheeting on the ground and water infiltration in the soil.

## 2. Materials and Methods

### 2.1. Study Site

This study was performed during the dry season from February to March 2016 in the forest of the Jubilee Garden of the Indian Institute of Science (13°01′17′′ N and 77°34′14′′ E) in Bangalore city, Karnataka state, India. This ecosystem has a tropical savannah climate with distinct wet and dry seasons, and the annual rainfall ranges from 900 to 1100 mm yr^−1^ [22,31]. The soil is described as an Alfisol based on U.S. Soil Taxonomy (United States Department of Agriculture, USDA) or a Luvisol according to Food and Agriculture Organization (FAO) classification. The first soil layer (0–10 cm depth) is mainly sandy (≈60%) and contains approximately 10 to 20% clay, mainly kaolinite [32]. The soil pH is 5.7, and its C content reaches 2.2% on average [22]. The vegetation is a deciduous forest dominated by acacia trees, mainly *Acacia auriculiformis*.

In this study site, the litter-feeding termites are mainly *Odontotermes feae*, *O. obesus* and *O. feoides,* and their activity involves the production of soil sheetings on wood logs or fallen leaves on the ground [22,31]. *Odontotermes* spp. belong to the Macroterminae subfamily, also named fungus-growing termites. If *Odontotermes* spp. may appear as major crop pests in some environments, they also play an important role in more natural environments where they act as key decomposers and bioturbators.

### 2.2. Experimental Design

Repacked soil cores were prepared using plastic cylinders (PVC) (5 cm in height and 15 cm in diameter). The soil was sampled from the 0–10 cm top-soil layer, air dried and sieved through a 2 mm mesh before being compacted in the cylinders until reaching a density similar to that found in the field (i.e., 1.2 g cm^−3^). In total, each cylinder contained 500 g of soil (dry weight, DW). Cylinders were inserted into the soil to a depth of 3 cm at randomly chosen locations (mean average distance between samples: ≈2 m). The soil in the cylinders was covered by organic resources, namely leaves of elephant grass (*Pennisetum purpureum*: “EG”, 16 g DW) or acacia (*Acacia auriculiformis*: “AL”, 11 g DW), elephant dung pats (*Elephas maximus*: “ED”, 230 g DW) or 10 cm² layers of cardboard (“CB”, 14 g DW). These substrates were chosen because of their availability and attractiveness to termites (*n* = 25 with 4 treatments + 1 control without organic resource × 5 replicates).

### 2.3. Biodiversity and Food Consumption Rates

After two months, the soil macrofauna within or below the organic resources were sampled and preserved in 70% alcohol. Individuals were identified and their numbers were counted. Organic substrates were collected, air dried and weighed. The food consumption rate was calculated as the percentage of weight lost.

### 2.4. Termite Bioturbation

At the end of the experiment, soil sheetings that covered the organic resources were collected, dried at 40 °C for 2 days and weighed. The soil turnover activity was measured by dividing the dry weight of sheetings by the quantity of organic material removed [26]. The number and diameter of galleries open on the surface were also measured with a caliper. Soil hydraulic conductivity at saturation (*K_sat_*) was measured with the Beerkan method [33,34,35]. A fixed volume of water (100 mL) was poured into the cylinder, and the time needed for the water to infiltrate was measured. The procedure was repeated between 7 to 10 times until a steady state of infiltration was reached. The soil cores (≈530 cm^3^) were then used to determine the soil bulk density, ρ (g cm^−3^) and the initial volumetric water content, θ_i_ (m^3^ m^−3^), was measured after sampling the surrounding top-soil environment (0–5 cm depth). The results were analysed with the original BEST algorithm [35].

Root biomass was measured in the soil cylinders. Samples were placed in beakers and soaked in water for at least 30 min, such that the soil aggregates could be easily broken down, after sieving through a 200 μm mesh. Roots were rinsed to remove soil particles and dried at 60 °C for 48 h.

### 2.5. Statistical Analyses

The normality of residuals was tested using the Shapiro–Wilk test. One-way analysis of variance (ANOVA) and least significant difference (LSD) tests were performed to assess differences between means. Partial least squares regression (PLSR) analysis was used to identify important variables linked to soil hydraulic conductivity. Correlations between variables were tested using the Pearson method. All statistical calculations were carried out using R version 3.5.1 (https://www.r-project.org/). Differences among treatments were declared significant at the < 0.05 probability level.

## 3. Results and Discussion

### 3.1. Organic Resource Consumption by Termites

**Figure 1:**
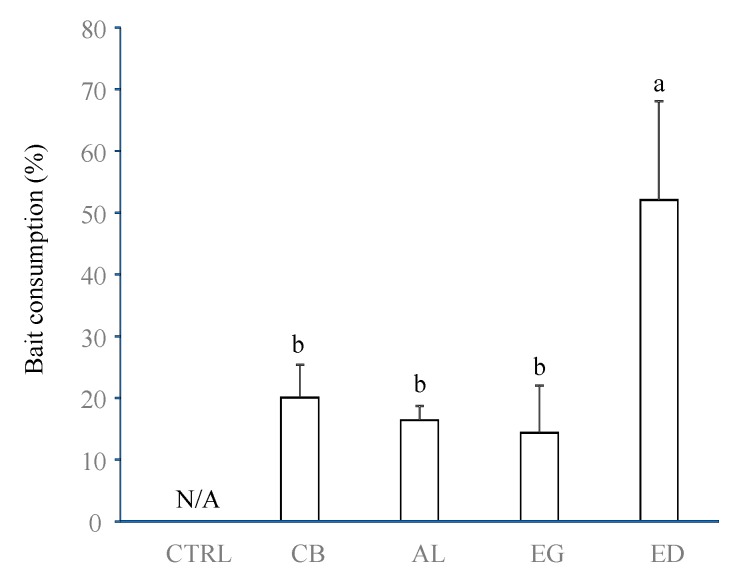
Rate of organic resource consumption by termites, in % of the initial organic resource weight loss (elephant dung (“ED”), acacia leaves (“AL”), elephant grass (“EG”) and cardboard (“CB”)), Error bars represent the standard error of the mean. Histograms with the same letter are not significantly different at *p* ≥ 0.05 (n = 5). N/A for non-applicable in the case of the control.

At the end of the experiment, termites were only found in or beneath the elephant dung pads. A very small amount of earthworm casts was found beneath two cardboard pieces. Termite individuals were all identified as belonging to *O. obesus* (Termitidae, Macrotermitinae), thus confirming the importance of this termite species in southern-Indian ecosystems [17]. Termite occurrence was associated with a consumption of the organic material, as shown by the more important consumption rate of ED than the other organic resources (Figure 1, ANOVA test, *F_4,20_* = 5.30, *p* = 0.005), while no differences occurred between the AL, EG and CB treatments (*p* > 0.05 between each pair).

The opportunistic consumption of mammalian dung by termites has been described in Africa for a long time [36,37]. Our study is, however, the first to stress the higher attractiveness of elephant dung to *O. obesus* in Asia compared to other organic resources. The rapid detection and consumption of ED by termites is in line with Cheik et al. [38], who found that elephant dung can be used to stimulate termite foraging activity in southern India. The low attractiveness of EG, CB, and AL to termites was more surprising since cellulose baits are commonly used to measure termite foraging activity in dry regions [39,40] and because termites respond the most to substrates with the highest C:N ratio [41]. We explain this low activity in EG, CB and AL treatments by the high attractiveness of ED in terms of nutrient content and/or because it provides a thermal shadow [42]. However, our experiment only lasted for 2 months, and it is likely that these substrates would have been more consumed if the experiment had lasted longer. Consequently, it can be concluded from this experiment that elephant dung is a sporadic and unpredictable but effective resource for termites and might be preferred over plant litter (EG and AL) or only cellulose (CB).

### 3.2. Relationship between Feeding Preferences and Soil Functioning

**Figure 2:**
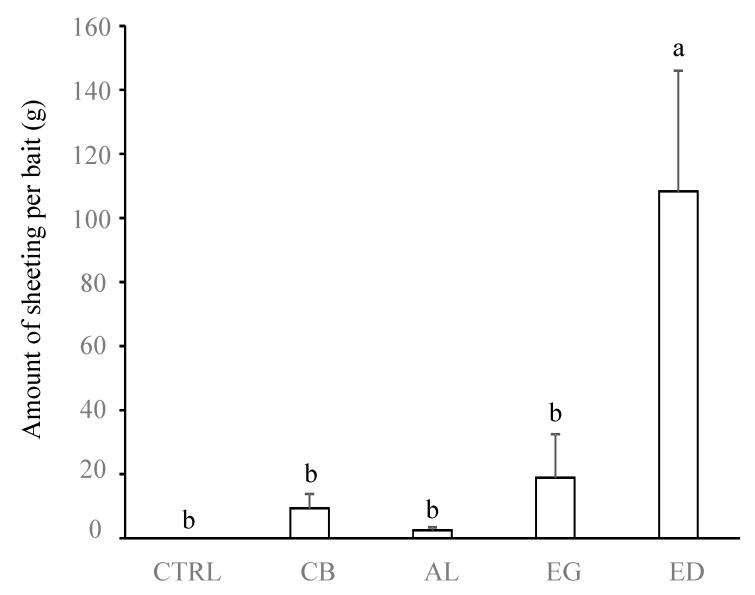
Amount of termite sheeting (g) for each treatment. Treatments are: control (“CTRL”), elephant dung (“ED”), acacia leaves (“AL”), elephant grass (“EG”) and cardboard (“CB”). Error bars represent the standard error of the mean. Histograms with the same letter are not significantly different at *p* ≥ 0.05 (n = 5).

**Figure 3:**
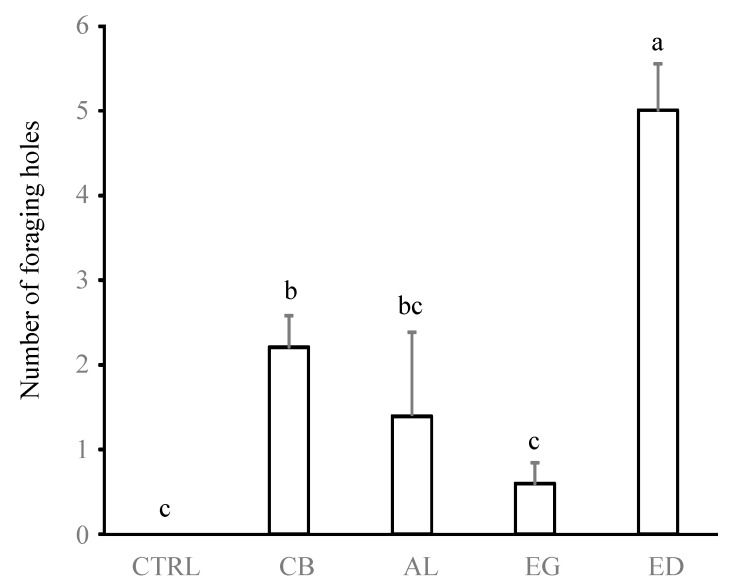
Number of foraging holes observed on the soil surface. Treatments are: control (“CTRL”), elephant dung (“ED”), acacia leaves (“AL”), elephant grass (“EG”) and cardboard (“CB”). Error bars represent the standard error of the mean. Histograms with the same letter are not significantly different at *p* ≥ 0.05 (n = 5).

**Figure 4:**
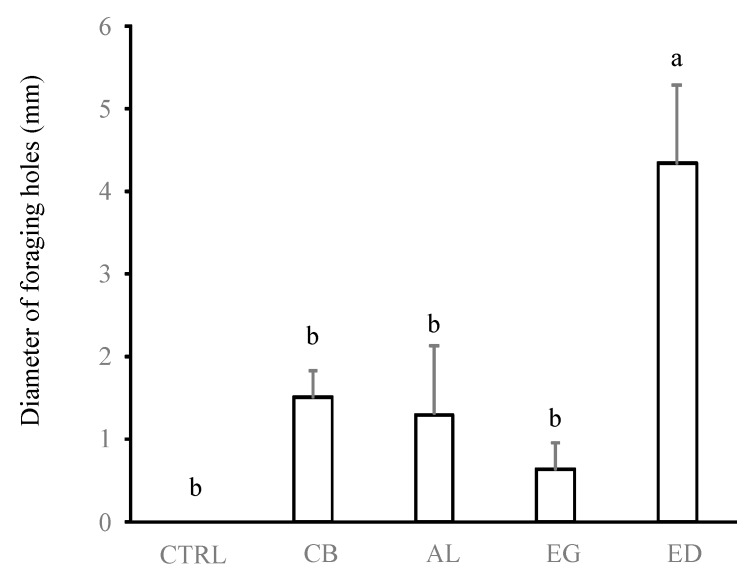
Average diameter of foraging holes (in mm). Treatments are: control (“CTRL”), elephant dung (“ED”), acacia leaves (“AL”), elephant grass (“EG”) and cardboard (“CB”). Error bars represent the standard error of the mean. Histograms with the same letter are not significantly different at *p* ≥ 0.05 (n = 5).

Figure 2, Figure 3 and Figure 4 show that the preference of termites for ED was associated with a more important production of soil sheeting and more numerous and larger pores at the soil surface compared with the other substrates (*p* < 0.05 in all cases, Table 1). Conversely, no significant differences in sheeting production and diameter of the foraging holes were found between the AL, EG, CB and CTRL treatments (*p* > 0.05 in all cases), while the numbers of foraging holes in the CB and AL treatments were intermediate to those for the ED, EG and CTRL treatments.

**Figure 5:**
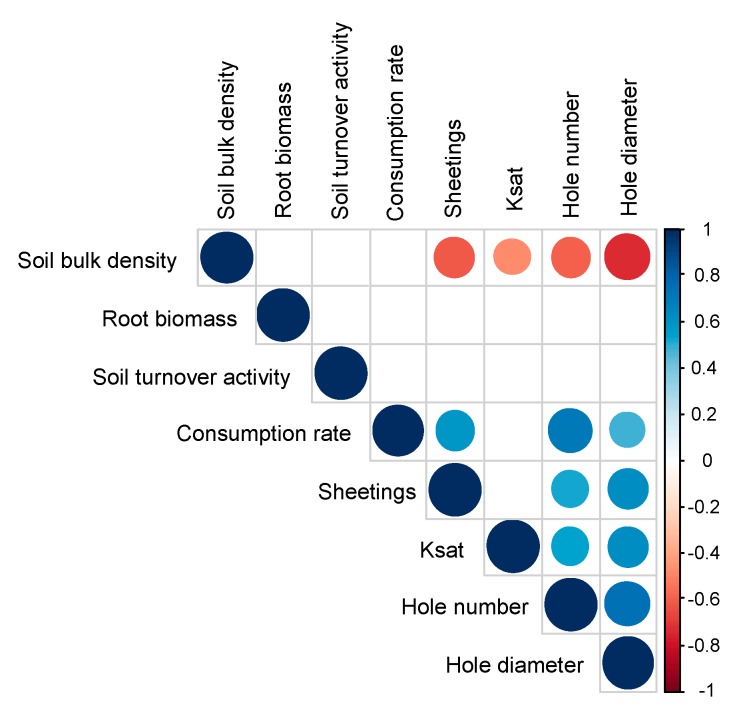
Matrix of correlations between variables. Only significant correlations (*p* < 0.05) are given. The scale colour indicates whether correlations between variables were positive (closer to 1, blue circles) or negative (closer to −1, red squares).

These findings are clearly in line with those from studies carried out in Africa [25,43,44,45] that showed that fungus-growing termites bring soil from different soil layers to build the sheetings that they use to cover themselves and the food they consume. Although the specific chemical, physical and biological properties of termite sheetings have been previously described [23], demonstration of the relationship between termite feeding preferences and the production of sheetings above-ground or the construction of galleries below-ground remains limited to studies that have been carried out in Africa [25,46,47]. In our study, no significant difference in soil turnover activity was measured between treatments (Table 1), suggesting that the returns on investment (i.e., the amount of energy spent on building the sheeting per the amount of energy received from the organic resources) were similar despite the difference in food quality and preference. However, the production of sheeting was positively related to the consumption rate and the diameter of foraging holes (Figure 5).

**Figure 6:**
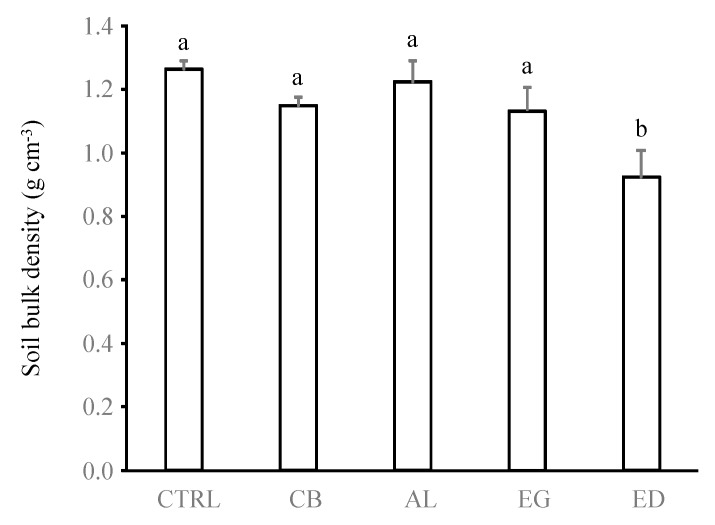
Soil bulk density (g cm^−3^) below the treatments (control (“CTRL”), elephant dung (“ED”), acacia leaves (“AL”), elephant grass (“EG”) and cardboard (“CB”)). Error bars represent the standard error of the mean. Histograms with the same letter are not significantly different at *p* ≥ 0.05 (n = 5).

Despite the fact that these results have to be interpreted with caution because variables reached important values only for the ED treatment but remained very low for the other treatments, the findings of our study suggest that the amount of sheeting can be used as an indicator of *O. obesus* feeding preference. These findings also suggest that the more *O. obesus* termites prefer a substrate, the more they circulate within galleries and, as a consequence, increase the diameters of the gallery openings. This hypothesis explains the significant reduction in soil bulk density in the ED treatment compared with the other treatments (*p* < 0.05 in all cases, Figure 6), as well as the negative relationship of soil bulk density with the diameter of foraging holes and the quantity of soil sheeting (Figure 5).

**Figure 7:**
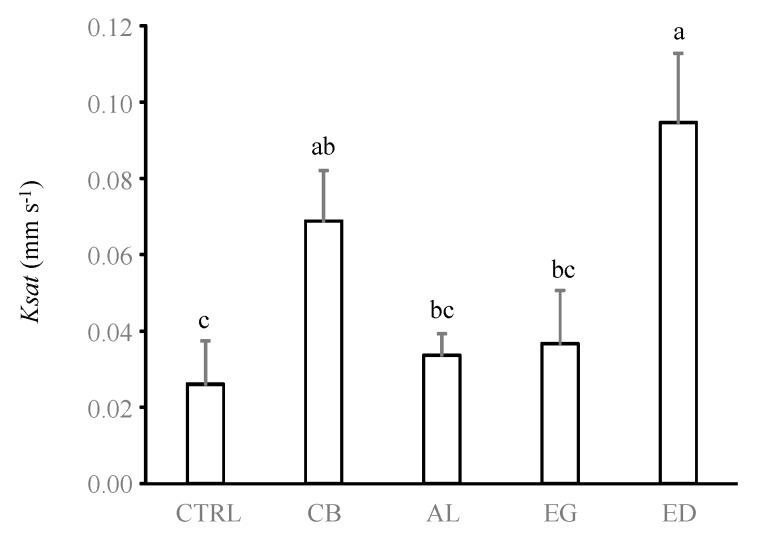
Soil hydraulic conductivity (*Ksat*, in mm s^−1^) for the different treatments (control (“CTRL”), elephant dung (“ED”), acacia leaves (“AL”), elephant grass (“EG”) and cardboard (“CB”)). Error bars represent the standard error of the mean. Histograms with the same letter are not significantly different at *p* ≥ 0.05 (n = 5).

*Odontotermes* spp. mainly build superficial and horizontal galleries localized in the first centimetre of the soil [31,47]. Our study shows that the production of these foraging galleries was associated with a reduction in soil bulk density in the ED treatment as well as an increase in *K_sat_* of 4 and 2.6-fold in the ED and CB treatments compared with the CTRL treatment, respectively (ANOVA test, *F*_4,10_ = 8.36, *p* = 0.003, Figure 7). Conversely, no significant differences were measured in the AL, EG and CTRL treatments (*p* > 0.05). These results are in line with those found in Cheik et al. [31] (3-fold) and Kaiser et al. [26] (1.5 to 9.28-fold), although termite foraging is usually increased by only 1.5- to 3-fold [27,47,48].

The diameter and number of holes were identified as the most important factors associated with *K*_sat_ (PLSR model, Q^2^ = 0.52, Root Mean Square Error of Prediction (RMSEP) = 0.042, Table 2), which is in line with the general assumption that a higher soil macroporosity increases the soil hydraulic conductivity [49].

Consequently, the findings of this study confirmed the general assumption that the stimulation of termites can accelerate the infiltration of water in soil, mainly through the production galleries that are open on the soil surface [26,27,28,47,50].

## 4. Conclusions

Although a large number of studies have reported a link between termite feeding preferences and soil bioturbation in Africa, this relationship remains poorly understood in Asia where services provided by termites can differ, for instance due to different soil, climate and termite assemblage. This study is the first to stress that (i) the more termites prefer a substrate, the more they impact soil dynamic and water infiltration in the soil; (ii) elephant dung is an efficient amendment for stimulating termite activity in southern India; and (iii) its consumption by termites is associated with an increase in soil dynamic and water infiltration in the soil. In conclusion, we argue that an economic and agronomical study is now needed to determine whether this substrate can be used in cultivated agro-systems to attract termites and promote soil translocation, aeration and water infiltration.

## Figures and Tables

**Table 1:** Results of the ANOVA test showing the influence of organic resource quality (control, elephant dung, elephant grass, acacia leaves and cardboard) on the food consumption rate (%), diameter (mm) and number of foraging holes, amount of sheetings (g), soil bulk density (g cm^−3^), soil turnover activity (g soil g consumed^−1^) and root biomass (mg g^−1^) (n = 5 in all cases).

	*F_4,20_*	*p*-Value
Consumption rate	5.29	0.004
Diameter of holes	7.78	<0.001
Number of holes	13.04	<0.001
Amount of sheetings	6.37	0.004
Soil bulk density	4.59	0.009
Turnover activity	1.63	0.207
Roots biomass	1.80	0.168

**Table 2:** Variable important in the projection (VIP) scores from the most relevant variables used for the PLSR describing the evolution of soil hydraulic conductivity at saturation (*K_sat_*).

Variables	VIP Scores
Amount of sheetings	6.97
Decomposition rate	0.99
Hole diameter	0.02
Hole number	0.01
